# The IBD-disk accurately assesses disability and psychological burden at IBD diagnosis and predicts adverse outcomes in both UC and Crohn’s disease during the first year of treatment: a prospective observational cohort study

**DOI:** 10.3389/fgstr.2025.1642061

**Published:** 2025-09-11

**Authors:** Peter Rimmer, Viorelia Stoica, Maryam Ibrahim, Asima Javed, Karl Hazel, Michael Owusu, Daniel Regan-Komito, Rachel Cooney, Asif J. Iqbal, Iain Chapple, Philip Harvey, Tariq H. Iqbal

**Affiliations:** ^1^ Department of Gastroenterology, University Hospitals Birmingham NHS Foundation Trust, Birmingham, United Kingdom; ^2^ Institute of Microbes, Infection and Microbiomes, College of Medical and Dental Sciences, University of Birmingham, Birmingham, United Kingdom; ^3^ Department of Gastroenterology, Beaumont Hospital, Dublin, Ireland; ^4^ Roche Pharma Research and Early Development, F. Hoffmann-La Roche, Basel, Switzerland; ^5^ Institute of Cardiovascular Sciences, College of Medical and Dental Sciences, University of Birmingham, Birmingham, United Kingdom; ^6^ School of Dentistry, College of Medical and Dental Sciences, University of Birmingham, Birmingham, United Kingdom; ^7^ Department of Gastroenterology, Royal Wolverhampton NHS Trust, Wolverhampton, United Kingdom

**Keywords:** ulcerative colitis, Crohn’s disease, patient report outcome measure (PRO), inflammatory bowel disease, IBD disk, disability, psychological distress, mental health disorder

## Abstract

**Background:**

Inflammatory bowel disease (IBD) is linked with increased prevalence of mental health disorders (MHD), particularly anxiety and depression. How this influences treatment outcomes in the first year after diagnosis is poorly studied. The IBD disk is a patient-reported outcome measure that quantifies disease-associated disability. Our objectives were to determine if the disk can identify those at risk of adverse treatment outcomes during the first year after diagnosis and assess if it could accurately screen for significant mental health symptoms at IBD presentation.

**Materials and methods:**

Patients with suspected IBD were seen in a rapid-access clinic. An IBD disk was completed upon first review, pre-diagnosis. A subgroup simultaneously completed the Hospital Anxiety and Depression scale (HADS). Repeat disks were completed after diagnosis, with 12-month outcomes collected prospectively.

**Results:**

188 patients completed a baseline IBD disk (97 Crohn’s disease [CD], 91 Ulcerative colitis [UC]), 95 completed a simultaneous HADS and 82 completed a repeat disk after diagnosis and treatment. Pre-existing MHD were more frequent in CD. Pre-diagnosis, the IBD Disk ‘Emotions’ domain correlated with HADS depression (r_s_=0.607 p<.001), anxiety (r_s_=0.586 p<.001) and reliably identified HADS defined moderate-severe depression (Area under the curve [AUC] 0.873, 95% CI 0.804 – 0.942). An ‘Emotions’ domain score ≥7 identified all patients meeting this HADS threshold (Sensitivity 100%, specificity 60.5%, Youden’s index 0.601). The strength of discrimination fell post diagnosis (AUC 0.712, 95% CI 0.491 – 0.932), with ongoing high ‘Emotions’ domain scores strongly linked to disease activity in both CD and UC. Elevated baseline disk scores in UC predicted the subsequent need for advanced therapies (p=0.019), persistent active disease at 12 months (p=0.023) and need for inpatient treatment (p<.001). In CD, elevated disk scores predicted need for advanced therapies (p=0.014) and persistent active disease (p=0.015), though an association with the need for surgical resection within 12 months was not statistically significant (p=0.064).

**Conclusions:**

The IBD disk reliably screens for symptoms of depression and anxiety and identifies risk of adverse treatment outcomes at IBD presentation. Particularly in UC, higher disk scores at diagnosis could complement existing tools to better identify those who would benefit from early treatment escalation.

## Introduction

There is a high prevalence of mental health disorders (MHD) in those with established Inflammatory bowel disease (IBD). Two large meta-analyses have focussed on anxiety and depression, finding anxiety disorder present in 21% (but anxiety symptoms in up to 35%) and depressive disorder in 15% (with depressive symptoms in up to 25%) (1, 2). Comparatively, when last surveyed 17% of the United Kingdom (UK) general population met criteria for common mental health disorders (3). Both studies found a higher prevalence of depression and anxiety symptoms in those with Crohn’s disease (CD) than Ulcerative colitis (UC) and those with active disease over remission. Links have also been established between IBD and broad range of other MHD including deliberate self-harm, eating disorders, bipolar disorder and post-traumatic stress disorder (4–6). The relationship between IBD and MHD can be viewed as bi-directional. Both anxiety and depression are associated with higher rates of treatment escalation, hospitalisation and emergency service utilisation amongst IBD patients, whilst active IBD is linked to the future development of anxiety or depression (7, 8). A large primary care study has highlighted this amongst UK IBD populations (9). It has been demonstrated that targeted psychological interventions, where a need is identified, can have a positive impact on IBD associated outcomes (10).

Though attempts at determining the prevalence of MHD at the first presentation of IBD have been limited, the UK IBD standards group make clear the need for psychological assessment in the initial management of patients newly diagnosed with IBD (11, 12). A 2023 review focussing on the practicality of delivering psychological support in established IBD patients highlighted the time constraints placed upon outpatient clinics (13). The authors recommended the use of psychometric questionnaires such as the Hospital Anxiety and Depression Scale (HADS), which can take up to 5 minutes to complete (14). Time is often limited in new patient clinics and diagnostic uncertainty can increase the burden of anxiety. The option to utilise a tool that combines assessments of disease activity, disability and mental health burden is appealing. The IBD Disk (**Supplementary Appendix 1**) was first developed in 2017 (15). It represents a shortened self-administered version of the IBD Disability Index (IBD-DI) (16). The IBD-DI was developed as a multi-organisation cooperation based on four preparatory studies, a consensus conference and a subsequent operationalisation process (16). Conversely, the IBD disk followed an abridged development process and arose from an iterative Delphi consensus based upon opinion from selected gastroenterologists attending an industry funded training programme. Nonetheless, it was developed specifically with the ‘busy outpatient clinic’ in mind. It is deliberately broad in scope and employs a visual analogue scale. It has been validated as a measure for daily life burden in a large multicentre study (17). Disability as measured by the IBD disk has been shown to correlate well with the IBD-DI, as well as with C-reactive protein (CRP) and faecal calprotectin (FCP) (18). More recently, albeit on a smaller scale, multiple disk domains have been shown to correlate with disease activity in ileal CD, as measured by bowel wall thickness on abdominal ultrasound (19). Though it is reasonable to extrapolate these findings to disease onset, the utility of the IBD disk as a measure specifically of psychological disease burden, has not been validated. The HADS score is a long-established validated measure to screen for major depression and anxiety amongst individuals with physical health problems (20, 21). As such, it represents a suitable comparator for other scoring systems.

The primary objective for this work was to determine if disease associated disability at first presentation, as determined by the IBD disk, could be used to predict outcomes during the first year of treatment. This included the need for treatment escalation, the presence of persistent disease activity and the need for inpatient admission. A secondary aim, carried out as a sub-study, was to determine if the IBD disk could be utilised at first presentation to screen for clinically significant mental health symptoms.

## Materials and methods

Between February 2021 and June 2024, patients were triaged to a dedicated rapid access clinic for suspected IBD. Triage was based on having symptoms broadly compatible with IBD and an elevated (no mandated threshold) faecal calprotectin (FCP) level. The collection of the established and validated clinical indices and patient reported outcome measures (PROM) employed in this study took place as part of a wider prospective observational cohort study to which patients provided informed consent (IRAS 287279, approved by Bloomsbury Research Ethics Committee [REC reference 21/PR/0515]). During the first outpatient appointment, prior to the establishment of a diagnosis, patients were asked to complete an IBD disk score. This was completed in the clinic room, following discussion regarding the suspected diagnosis and investigative plan. A subgroup of patients were also asked to complete a simultaneous HADS score. Traditional clinical and biochemical indices, symptom duration and longitudinal treatment outcomes were collected prospectively. Repeat scores were taken at follow up attendances. Treatment was not protocolised for this study, but all patients were managed by the same multi-disciplinary group of IBD clinicians within a single hospital and treatment commenced on the day of colonoscopy where endoscopic findings were supportive. Pre-existing mental health diagnoses were obtained from clinical history taking and coded primary care diagnoses. IBD disk scores from patients in whom IBD was subsequently excluded have been omitted from the analysis.

At each follow-up visit, current treatments were recorded and an assessment of disease activity undertaken. Diagnoses of IBD were established in line with European Crohn’s and Colitis Organisation guidelines (22). The criteria utilised to define ‘Inactive’ disease are shown in **Supplementary Appendix 2**. A hierarchy of importance was employed in decision making. Where clinical indices alone were available these were used. If both CRP and FCP were available, these were preferred over clinical symptoms. If an endoscopic or radiological assessment had been performed, it superseded both clinical and biochemical indices.

Presented statistical analyses were primarily undertaken in JASP version 0.183 (23). Non-parametric tests were undertaken for all IBD disk related analyses as both the total IBD disk score (Shapiro-Wilk W 0.981 p=0.012) and each individual domain score did not follow a normal distribution. The Mann-Whitney U (U) test has been utilised for grouped differences in continuous variables. A chi-squared (X^2^) test is employed to determine proportional difference in categorical variables. For receiver operating characteristic (ROC) curves and logistic regression modelling, Jamovi version 2.6.26 was utilised alongside a dedicated R package and integrated modules (24–27).

## Results

IBD disk scores were collected from 188 patients (97 CD, 91 UC) at their first outpatient appointment, prior to diagnosis. 95 (45 CD, 50 UC) completed a simultaneous HADS score. 128 patients seen via this pathway completed an IBD disk and subsequently had IBD excluded. Follow up IBD disk scores were collected post treatment (median interval 117 days) from 82 patients, of whom 37 completed a paired post-treatment HADS score. The cohorts contributing to each analysis are shown in **Table 1**.

**Table 1:** Cohort descriptions for each subgroup contributing to presented analyses.

Total IBD disk visit 1 cohort			
	Crohn’s	UC	Global test
N	97	91	
Age median (IQR)	28 (15)	33 (17)	U=3716 p=0.06^1^
Sex (% male)	41%	58%	X^2^ 5.43 p=0.020^2^
Baseline FCP, median (IQR), ug/g	791 (1341)	1479 (1342)	U=2731 p=0.001^1^
Disease extent/location/non-IBD type (%)	Ileal: 47 (48)	Proctitis: 25 (27)	
	Colonic: 20 (21)	Left sided: 28 (31)	
	Ileocolonic: 30 (31)	Extensive: 38 (42)	
Pre-existing MHD (% yes)	22%	11%	X^2^ 3.88 p=0.049^2^
Current antidepressants (% yes)	16%	7%	X^2^ 4.45 p=0.035^2^
Current antipsychotics (% yes)	3%	0%	X^2^ 2.86 p=0.091^2^
Cohort providing a paired HADS at visit 1			
N	45	50	
Age median (IQR)	28 (12)	31.5 (17.75)	U=1025 p=0.46^1^
Sex (% male)	38%	56%	X^2^ 3.15 p=0.076^2^
Baseline FCP, median (IQR), ug/g	721 (1276)	1480 (1714)	U=756 p=0.039^1^
Disease extent/location/non-IBD type (%)	Ileal: 22 (49)	Proctitis: 16 (32)	
	Colonic: 11 (24)	Left sided: 15 (30)	
	Ileocolonic: 12 (27)	Extensive: 19 (38)	
Pre-existing MHD (% yes)	27%	14%	X^2^ 2.38 p=0.123^2^
Current antidepressants (% yes)	22%	12%	X^2^ 1.76 p=0.18^2^
Current antipsychotics (% yes)	4%	0%	X^2^ 2.27 p=0.13^2^
Cohort providing a repeat IBD Disk			
N	49	33	
Age median (IQR)	27 (12)	33 (11)	U=561 p=0.020^1^
Sex (% male)	40%	60%	X^2^ 3.76 p=0.052^2^
Baseline FCP, median (IQR), ug/g	720.5 (852.75)	1949 (1338)	U=376 p<.001^1^
Disease extent/location/non-IBD type (%)	Ileal: 23 (47)	Proctitis: 5 (15)	
	Colonic: 10 (20)	Left sided: 14 (42)	
	Ileocolonic: 16 (33)	Extensive: 14 (42)	
Pre-existing MHD (% yes)	24.5%	12%	X^2^ 1.92 p=0.166^2^
Current antidepressants (% yes)	16%	6%	X^2^ 1.94 p=0.164^2^
Current antipsychotics (% yes)	4%	0%	X^2^ 1.38 p=0.240^2^
Visit 2 FCP median (IQR), ug/g	218 (644)	211.5 (272.75)	U=335 p=0.444^1^
Duration between visits (days)	114 (124)	118 (119)	U=725 p=0.628^1^

^1^Mann-Whitney ^2^Chi-Squared X^2^.

The CD cohort was characterised by a significantly higher proportion of pre-existing MHD than those with UC (CD 22% [21/97], UC 11% [10/91], X^2^ 3.88 p=0.049). Including those with multiple diagnoses; depression, anxiety disorder and mixed anxiety and depression were the most frequent MHD, accounting for 73% (27/37) of all pre-existing MHD. The most frequent anti-depressant prescribed was Sertraline (9 [41% of all antidepressants]) followed by Citalopram (4 [18%]).

### Quantifying disability and psychological disturbance at baseline

The overall IBD disk score was higher in those subsequently diagnosed with CD (median 56, IQR 30), relative to UC (median 45, IQR 36.5, Mann-Whitney p=0.002). The individual scores for each domain are presented, split by diagnosis, in **Table 2**. This increase in disease-associated disability was driven by significantly higher scores across multiple IBD Disk domains including *‘Abdominal pain’* (p<.001), *‘Interpersonal interactions’* (p=0.04), *‘Energy’* (p<.001), *‘Emotions’* (p=0.02) and *‘Body image’* (p<.001). A binomial logistic regression was performed to identify the key drivers of the differences observed. The association of all 10 IBD disk domains with a CD or UC diagnosis was modelled, with UC coded as ‘Class 1’. The overall model was significant (Overall model test X^2^ 29.9 p<.001). The only factor significantly associated with a UC diagnosis was ‘*Regulating Defecation’* (Odds ratio 1.179 95% CI 1.055 – 1.318), whilst *‘Abdominal pain’* (Odds ratio 0.878 95% CI 0.788 – 0.978) and *‘Body Image’* (Odds ratio 0.874 95% CI 0.775 – 0.985) significantly favoured CD. Pre-existing MHD did associate with a higher baseline IBD disk score across IBD (Median, MHD=59, no MHD=49, U=1762 p=0.015). However, IBD subtype (t=2.8 p=0.006) had a far greater impact on total IBD Disk score than the presence of a MHD (t=1.86 p=0.064) when modelled together in a linear regression (overall model R 0.257, F 6.54, p=0.002).

**Table 2:** *Median (interquartile range)* IBD disk domain scores (entire cohort) at baseline, split by final diagnosis, with the HADS scores from the relevant subgroup at the end of the table.

Domain	Crohn’s	UC	Mann-Whitney U
IBD disk domains (n=188)			
Abdominal Pain	8 (4)	6 (5.5)	p<.001
Regulating Defecation	5 (7)	6 (8)	p=0.52
Interpersonal Interactions	3 (6)	2 (4.5)	p=0.04
Education and Work	6 (6)	4 (7)	p=0.11
Sleep	7 (5)	5 (7)	p=0.05
Energy	9 (3)	8 (4)	p<.001
Emotions	7 (4)	6 (5)	p=0.02
Body Image	6 (6)	3 (5)	p<.001
Sexual Functions	2 (7)	2 (6)	p=0.21
Joint Pain	5 (7)	2 (5.5)	P=0.04
HADS domains (n=95)			
Anxiety	8 (8)	7 (6)	p=0.58
Depression	6 (7)	4.5 (6.75)	p=0.17

### The impact of symptom duration at diagnosis on psychological disease burden

The presence of a pre-existing MHD did not associate with a different length of historical symptoms prior to first clinical assessment in CD (U=865 p=0.43) or UC (U=393 p=0.97). However, across IBD subtypes, a longer symptom duration positively correlated with overall IBD disk score (Spearman’s r_2_ = 0.153 p=0.039). Looking at individual IBD disk domains, IBD patients with an *‘Emotions’* domain score of ≥7 (out of 10) had a significantly longer symptom duration that those that did not (n=183, median 5 vs 9.5 months, U=5044 p=0.016). This is more pronounced in CD (n=95, median 6 vs 18 months, Mann-Whitney p=0.04). In CD, differences in symptom duration for those presenting with stricturing and penetrating complications did not reach significance (Montreal B1 n=80 median=9 months, B2/B3 n=15 median=24 months, Mann-Whitney p=0.08).

### The IBD Disk as a screening tool for significant mental health symptoms

**Figure 1:**
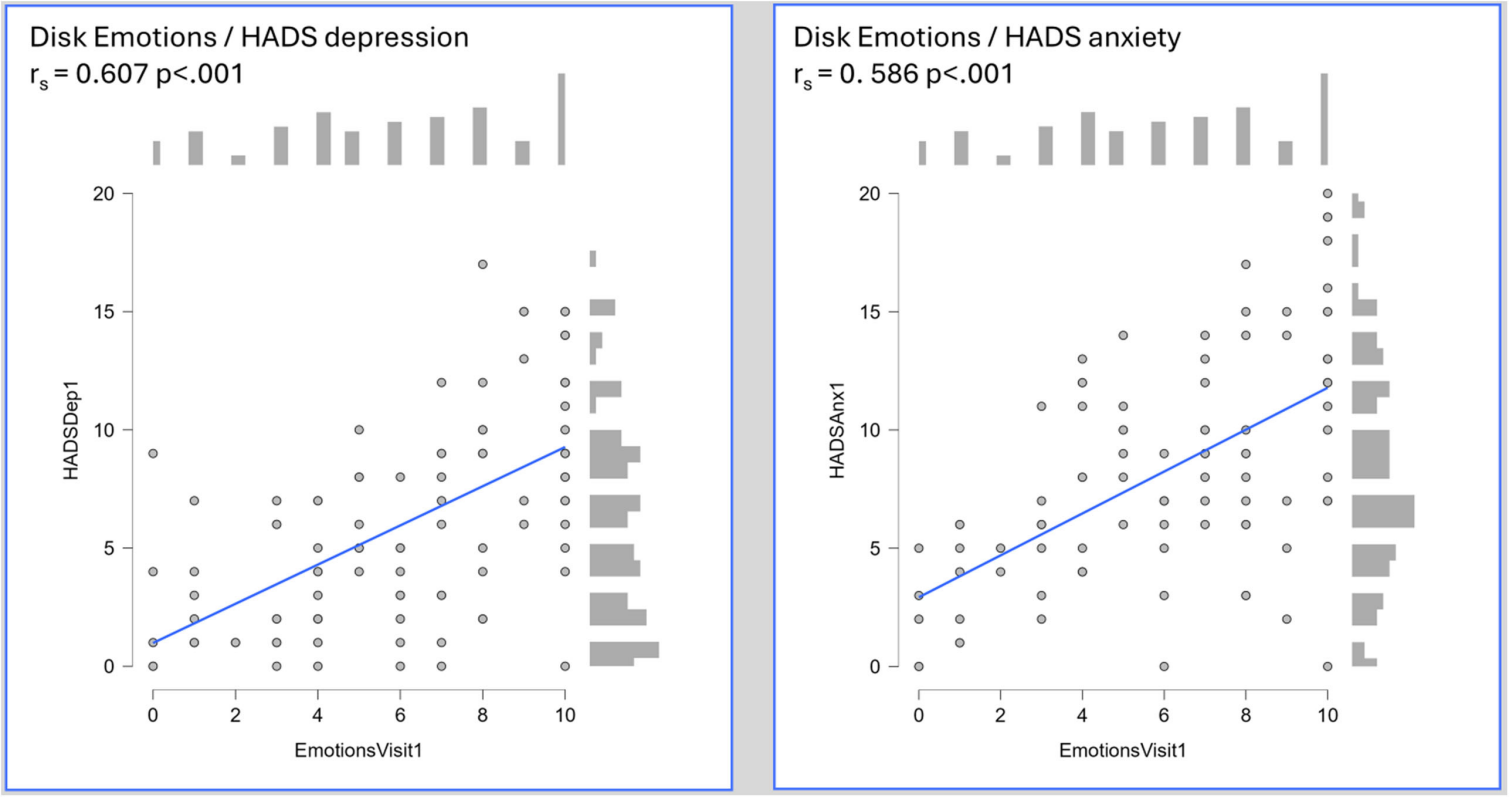
Heatmap of Spearman’s rank coefficients for correlation between individual disk domains and HADS anxiety and depression scores amongst 95 patients presenting with IBD (45 CD, 50 UC). Individual scatter plots are shown for the ‘Emotions’ domain.- An individual scatter plot of paired HADS depression and IBD disk *‘Emotions’* scores, with a histogram of score frequency around the outside of the plot. The correlation, as measured by Spearman’s rho, is highly statistically significant (HADS depression r_s_ 0.607 p<.001),- An individual scatter plot of paired HADS anxiety and IBD disk *‘Emotions’* scores, with a histogram of score frequency around the outside of the plot. The correlation, as measured by Spearman’s rho, is highly statistically significant (HADS anxiety r_s_ 0.586 p<.001).

Across IBD, strong correlations were seen between individual IBD disk domains and HADS scores for both depression and anxiety. For both HADS anxiety and depression scores, the *‘Emotions’* domain outperformed all others (**Figure 1**). Post treatment, during the second visit, IBD disk *‘Emotions’* scores remained most strongly associated with HADS Depression scores (n=36 r_s_ = 0.579 p<.001).

**Figure 2:**
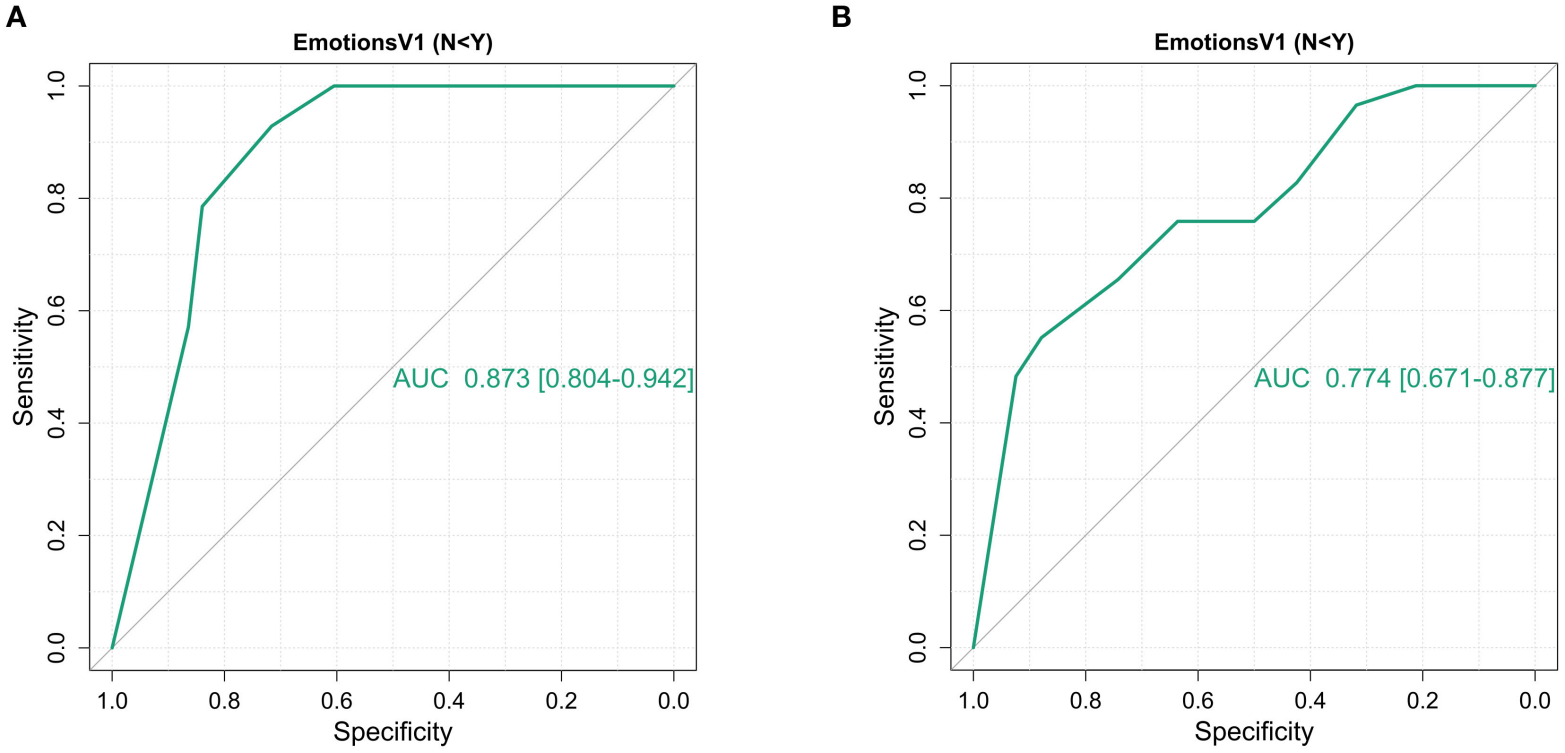
Receiver operating characteristic curves of the ability of the IBD disk ‘*Emotions’* domain to identify HADS depression and anxiety scores of moderate severity (≥11) amongst 95 patients at first presentation of inflammatory bowel disease. **(A)** Overall, the ‘*Emotions’* domain had an AUC of 0.873 [95% CI 0.804 – 0.942] for HADS depression scores ≥11.An *‘Emotions’* domain score ≥8 had the highest Youden’s index (0.645) but in a screening situation, a cut off ≥7 identified all individuals meeting the HADS cut off whilst retaining a high Youden’s index (Sensitivity 100%, specificity 60.5%, Youden’s index 0.605). **(B)** The *‘Emotions’* domain performed less well, both overall and at pre-specified cut offs for HADS anxiety, with an AUC of 0.774 [95% CI 0.671 – 0.877] for HADS anxiety scores ≥11. Here the optimal cut-off statistically was 9 (Youden’s index 0.431) but again a score of ≥7 would be favoured as it missed far few patients meeting the cut-off (Youden’s index 0.395 but sensitivity 75.8%, specificity 63.6%).

To validate the IBD disk, specifically the *‘Emotions’* domain, as a viable screening tool for clinically significant psychological symptoms, an appropriate cut off had to be sought. At least moderate HADS determined symptoms of depression and anxiety (score ≥11 for each) were thus sought. 15% (14/95) of patients met this threshold for HADS depression, of whom 36% (5/14) had an existing MHD. The *‘Emotions’* domain scores when plotted on a receiver operating characteristic (ROC) curve carried an area under the curve (AUC) of 0.873 (95% CI 0.804 – 0.942) for the HADS depression score cut-off. Statistically, the optimal cut point was a score of ≥8 (Sensitivity, 92.86%, specificity 71.6%, Youden’s index 0.645), though a score of ≥7 captured all of those with significant depression scores (Sensitivity 100%, specificity 60.49%, Youden’s index 0.605). For HADS anxiety, 30.5% (29/95) met the specified threshold, of whom 34% (10/29) had an existing MHD. The accuracy of the *‘Emotions’* domain was lower, with an overall AUC of 0.774 (95% CI 0.671 – 0.877). The optimal cut-point statistically was an *‘Emotions’* score of 9 (Sensitivity 55.17%, specificity 87.88%, Youden’s index 0.431) though a lower threshold of ≥7 again carried greater clinical relevance as a screening tool (Sensitivity 75.86%, specificity 63.64%, Youden’s index 0.395). The individual ROC plots of this data are shown in **Figure 2**. A binomial logistic regression model was developed to assess the predictive capacity of the *‘Emotions’* domain when adjusting for baseline patient and disease characteristics including age, sex, presence of an existing mental health diagnosis, haemoglobin, CRP and baseline faecal calprotectin. Due to missing FCP data, this model included 78 patients. The model for moderate depression scores was significant (Overall model test X^2^ 25.3 p=0.003) with an overall AUC of 0.910. The *‘Emotions’* domain score was the only independently significant predictor within the model (odds ratio 2.84 95% CI 1.38 – 5.85). Whilst model performance deteriorated for moderate anxiety scores, it remained significant (Overall model test X^2^ 24.2 p=0.004) with an overall AUC of 0.797. The *‘Emotions’* remained the only independently significant predictor (odds ratio 1.40 95% CI 1.09 – 1.78) within the model.

The utility of IBD Disk after diagnosis and treatment was interrogated in the 37 patients who went on the complete a further, post-diagnosis IBD Disk and HADS. This cohort was too small for modelling. Nonetheless, the unadjusted AUC fell to 0.712 (95% CI 0.491 – 0.932) for the depression cut-off and 0.707 (95% CI 0.463 – 0.952) for the anxiety cut off. The ≥7 threshold demonstrated inferior performance across both depression and anxiety scores (Sensitivity 57%, Specificity 70%, Youden’s index 0.271 for both).

### Drivers of ongoing disability and psychological symptoms after IBD diagnosis and treatment

Once the diagnosis has been confirmed and treatment initiated, the burden of symptoms for patients attending their first follow-up clinic was quantified. Across the cohort, this took place a median of 117 (IQR 126.25) days after the first pre-diagnosis clinic visit. In both CD and UC, the key driver of difference in the IBD disk score at the follow-up clinic was disease activity. As demonstrated in our earlier linear regression, unequal contribution across IBD subtypes drove the higher baseline disk scores in those with a pre-existing mental health diagnosis. Indeed, upon repeat completion of an IBD disk at the follow up clinic, presence of an existing MHD did not associate with differences in total disk score(No MHD = median IBD disk score 41 MHD present = median 50. Mann-Whitney p=0.31). Furthermore, there was no proportional difference in patients reaching an inactive disease state by first follow-up when stratified by the presence or absence of a MHD (X^2^ 0.047 p=0.828).

**Figure 3:**
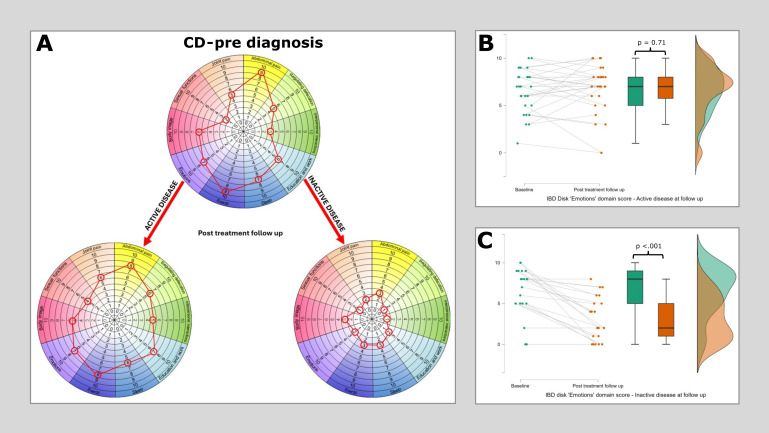
Median IBD disk scores for each domain in Crohn’s disease at baseline and then at post treatment follow-up, stratified according to disease state. **(A)** plots median IBD disk scores at baseline and post treatment follow up for 49 patients with CD, of whom 28 had active disease at follow up and 21 were inactive. Scores are plotted on the grid as would be aim when interpreting these on an individual patient basis in clinc. The only significant reduction identified in the ‘active’ disease group was in the ‘Energy’ domain (Pre diagnosis median 9, post diagnosis median 8, Wilcoxon 170 p=0.015). All observed differences in the ‘Inactive’ disease group reached statistical significance. **(B)** shows a raincloud plot of IBD disk ‘Emotions’ domain scores at baseline and at first follow up in those with persistent disease activity. There is no significant difference observed (Pre diagnosis median 7, post diagnosis median 7, Wilcoxon 148.5 p=0.71). **(C)** shows a raincloud plot of IBD disk ‘Emotions’ domain scores at baseline and at first follow up in those who reached an inactive disease state at this time point. A highly significant reduction is observed in this cohort (Pre diagnosis median 7, post diagnosis median 2, Wilcoxon 153 p<.001).

**Figure 4:**
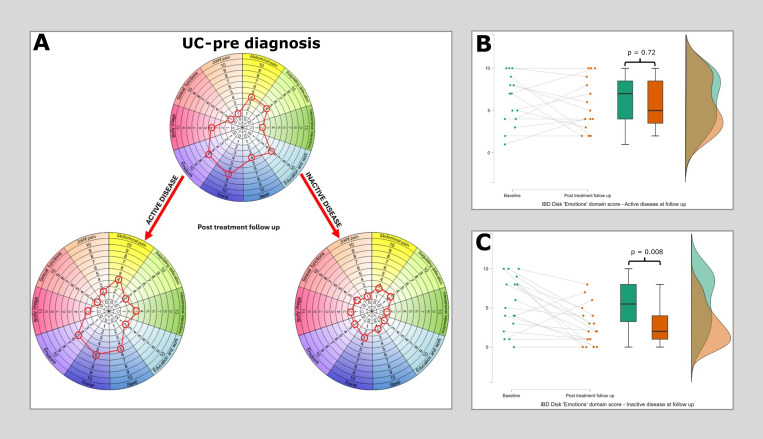
Median IBD disk scores for each domain in Ulcerative colitis at baseline and then at post treatment follow-up, stratified according to disease state. **(A)** plots median IBD disk scores at baseline and post treatment follow up for 33 patients with UC, of whom 15 had active disease at follow up and 18 were inactive. Scores are plotted on the grid as would be aim when interpreting these on an individual patient basis in clinc. The only significant reduction identified in the ‘active’ disease group was again in the ‘Energy’ domain (Pre diagnosis median 7, post diagnosis median 6, Wilcoxon 56 p=0.044). In the ‘Inactive’ disease group differences in the *‘Emotions’, ‘Abdominal pain’, ‘Regulated defecation’, ‘Education & work’, ‘Sleep’ and ‘Body image’* displayed statistically significant differences. **(B)** shows a raincloud plot of IBD disk ‘Emotions’ domain scores at baseline and at first follow up in those with persistent disease activity. There is no significant difference observed (Pre diagnosis median 7, post diagnosis median 7, Wilcoxon 37.5 p=0.72). **(C)** shows a raincloud plot of IBD disk ‘Emotions’ domain scores at baseline and at first follow up in those who reached an inactive disease state at this time point. A highly significant reduction is observed in this cohort (Pre diagnosis median 7, post diagnosis median 2, Wilcoxon 119.5 p=0.008).

When patients were stratified by whether they have reached an inactive disease state at first follow-up, a clear pattern emerges. Those with active disease carry a far higher overall IBD disk score in addition to scores across multiple domains (Post treatment IBD disk ‘active’ = 55 [median], Post treatment IBD disk ‘inactive’ = 27. Mann-Whitney p<.001). The median values at baseline and at the first post treatment follow up clinic, split by disease activity state, are shown for Crohn’s disease (**Figure 3**) and Ulcerative colitis (**Figure 4**). Additional focus is placed in these figures on the stark differences in the *‘Emotions’* domain. The cohort completing a repeat HADS score was underpowered (n=37), though scores were significantly higher in those with ongoing active disease compared to those in remission (HADS anxiety ‘active’ = 8 [median], ‘inactive’ = 6, Mann-Whitney p=0.02. HADS depression ‘active’ = 8, ‘inactive’ = 3, p=0.01).

### The IBD disk as a predictor of outcomes during the first 12 months of treatment

A total of 179 IBD patients (95 CD, 84 UC) had complete 12-month outcomes available. First, the impact of pre-existing MHD was quantified. CD patients with a pre-existing MHD received double the number of courses of oral steroids during this first year (MHD yes median=2, MHD no median=1, U=465.5 p=0.006). This did not reach significance in the smaller UC cohort of 8 patients with pre-existing MHDs (MHD yes median=1, MHD no median=0, U=238 p=0.312). Despite this, patients with pre-existing MHDs were no more likely to have ongoing disease activity at 12 months (overall X^2^ 2.87 p=0.09, UC X^2^ 1.12 p=0.29, CD X^2^ 0.77 p=0.38), progress to an advanced therapy (AT) within 12 months (overall X^2^ = 0.23 p=0.63, UC X^2^ 1.35 p=0.24, CD X^2^ 0.82 p=0.37) or require inpatient care in the first 12 months (overall X^2^ = 0.12 p=0.73, UC X^2^ 0.07 p=0.79, CD X^2^ 0.21 p=0.64).

**Figure 5:**
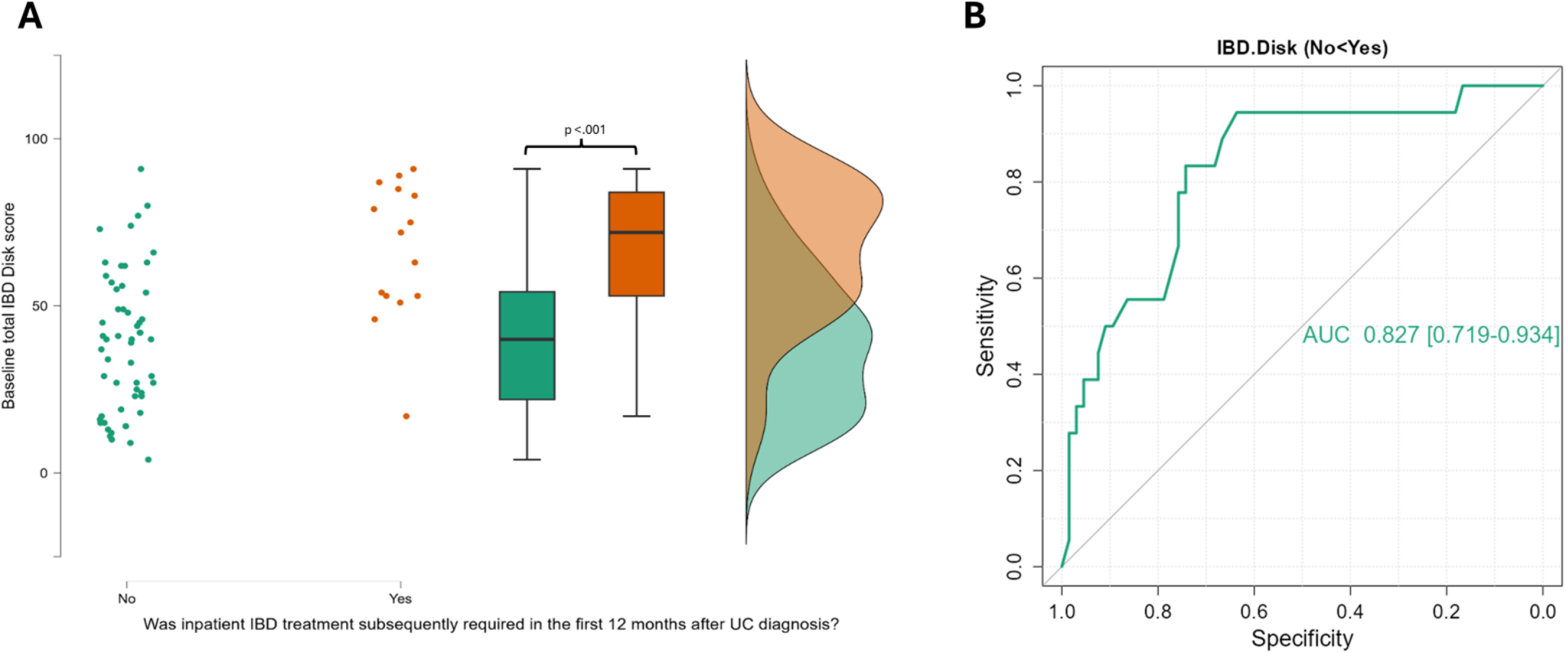
A raincloud plot demonstrating the significant elevation in IBD Disk scores at baseline in those with UC that go on to be admitted to hospital for IBD treatment in the first 12 months after diagnosis, alongside a ROC curve for all IBD disk scores. **(A)** This raincloud plot encompasses 84 UC patients of whom 18 required inpatient care for their colitis in their first 12 months after diagnosis. The median IBD Disk score at baseline was 40 (IQR 28.25) in those that did not required admission, and 66.5 (IQR 29) in those that did. This difference was highly significant on statistical testing (Mann-Whitney 206 p<.001). **(B)** The Area under the Curve for the ability of all IBD disk scores from UC patients to detect those that will need inpatient treatment in the first 12 months was 0.827 (95% CI 0.719 – 0.934). The optimal cut-point for this was a score of 45 (Sensitivity 94.4%, specificity 63.6%, Youden’s index 0.5.

Secondly the impact of baseline IBD disk scores and one-year outcomes was analysed. This is shown in **Table 3** with traditional clinical activity scores for comparison. A higher overall IBD disk score at baseline predicted an increased likelihood of progression to advanced therapy (AT) and having persistently active disease in both CD and UC. In UC, higher baseline IBD disk scores also predicted the need for inpatient IBD treatment during the following 12 months (**Figure 5**). In the underpowered cohort requiring bowel resection surgery, a trend was observed towards higher baseline IBD disk score being associated with needing a surgical resection within 12 months of CD diagnosis (U=177.5 p=0.06). This trend was not observed using the Harvey-Bradshaw index (U=246.5 p=0.51). Nonetheless, in both CD and UC, the disk had a lower AUC than traditional activity indices for detecting the need for AT, likelihood of persistent active disease and need for inpatient care (Partial mayo score and Harvey-Bradshaw index respectively). Finally, the total IBD disk score was observed to correlate with referral FCP in UC (r_s_=0.328 p=0.002) but not CD (r_s_=-0.02 p=0.74) and subsequent endoscopic severity at UC diagnosis (UCEIS r_s_=0.352 p=0.004) but not CD diagnosis (SESCD r_s_=0.157 p=0.16).

**Table 3:** Median (interquartile range), Mann-Whitney derived p values and AUC (95% CI) for all plotted values comparing the ability of the IBD disk and traditional disease activity index to differentiate CD and UC patients who will/will not require advanced therapies, surgical resections or inpatient care within the first 12 months, in addition to the rates of persistent disease activity at the 12-month timepoint.

Treatment Outcome	UC (n=84)		CD (n=95)	
	Partial mayo	IBD disk	Harvey bradshaw index	IBD disk
Advanced therapy within 12m *UC AT 18/84 (21%)* *CD AT 49/95 (52%)*	No AT: 4 (2)	No AT: 40 (31.5)	No AT: 6 (4)	No AT: 53 (29.25)
	AT: 7 (2)	AT: 54.5 (24.5)	AT: 9 (4)	AT: 65 (29)
	p<.001	p=0.019	p=<.001	p=0.014
	AUC 0.853 (0.744-0.962)	AUC 0.681 (0.539–0.824)	AUC 0.760 (0.658–0.862)	AUC 0.646 (0.534–0.758)
Active disease at 12m *UC Active 19/84 (23%)* *CD Active 37/95 (39%)*	Inactive: 4 (3)	Inactive 41 (33)	Inactive: 7 (5)	Inactive: 54 (32)
	Active: 6 (3)	Active: 54 (31.5)	Active: 9 (5)	Active: 62 (27)
	p=0.013	p=0.023	p=0.014	p=0.015
	AUC 0.689 (0.559 – 0.819)	AUC 0.672 (0.527–0.817)	AUC 0.653 (0.541–0.765)	AUC 0.649 (0.537–0.761)
Inpatient care during first 12m *UC IP care 18/84 (21%)* *CD IP care 15/95 (16%)*	No IP care: 3 (2)	No IP care: 40 (28.25)	No IP care: 7 (4)	No IP care: 56 (26.75)
	IP care: 7 (1)	IP care: 66.5 (29)	IP care: 9 (3.5)	IP care: 58 (26.5)
	p<.001	p<.001	p=0.199	p=0.366
	AUC 0.937 (0.885 – 0.989)	AUC 0.827 (0.719 – 0.934)	AUC 0.605 (0.466 – 0.744)	AUC 0.574 (0.412 – 0.736)
Resection within 12m *UC resection 2/84 (2%)* *CD resection 7/95 (7%)*	No resection: 4 (3)	No resection: 42 (34)	No resection: 8 (4)	No resection: 56 (26.75)
	Resection: 7 (0)	Resection: 59 (4)	Resection: 7 (4.5)	Resection: 77 (26.5)
			p=0.510	p=0.064
	Only 2 patients	Only 2 patients	AUC 0.576	AUC 0.712

In grouped IBD, a number of these trends can also be seen when looking at the ‘Emotions’ domain alone. If the previously discussed baseline threshold of an ‘Emotions’ score of ≥7 is again chosen, patients who met this received more oral steroid courses (U=4448 p=0.035), were more likely to progress to an advanced therapy within 12 months (X^2^ 3.96 p=0.047) and were more likely to require inpatient care during the first 12 months (X^2^ 7.21 p=0.007). There was a non-significant trend for higher baseline ‘*Emotions’* scores in those with persisting disease activity at 12 months (X^2^ 3.67 p=0.055).

## Discussion

The data presented signifies a novel evaluation of the IBD disk in a cohort of IBD patients assessed at the point of first presentation, prior to diagnosis. Whilst it is acknowledged that obtaining disk scores when diagnostic uncertainty remained may have heightened factors such as anxiety, our approach allowed for the quantification of baseline disease associated disability prior to any treatment being commenced and uninfluenced by any preconceptions about a given IBD type (28).

In IBD, disease associated disability has been shown to negatively impact upon health-related quality of life and socioeconomic productivity (29, 30). Disability determined by other PROM including the IBD-DI has been linked to adverse future treatment outcomes in both established and newly diagnosed IBD cohorts (31, 32). Moreover, normalised quality of life and the absence of disability now forms a long-term treatment target listed in STRIDE 2 (33). For the first time we have demonstrated that disability determined by the IBD disk can help identify both UC and CD patients at an increased risk adverse treatment outcomes in the first 12 months after diagnosis. The strength of association, particularly within UC where IBD disk scores also correlated with biochemical and endoscopic disease activity, highlights disability as an important marker of disease severity and treatment outcome. Whilst overall predictive capacity was not superior to traditional disease activity indices, our data supports the complementary role of the IBD disk as a PROM at IBD diagnosis across disease subtypes. Alongside these established markers, higher IBD disk scores at presentation should prompt consideration of a more aggressive therapeutic approach with earlier treatment escalation. In CD, whilst the cohort proceeding to surgical resection within 12 months was small, the disk was more able to identify these patients than the Harvey-Bradshaw index.

We have been able to demonstrate the high prevalence of significant psychological symptoms across IBD patients at presentation. This is particularly the case in patients subsequently diagnosed with CD, aligning with studies in those with established disease (1, 2). IBD disk scores, particularly in the *‘Emotions’* domain, strongly associated with HADS anxiety and depression scores. An *‘Emotions’* score of ≥7 identified all patients with HADS determined moderate or severe depressive symptoms at IBD presentation, but in many these symptoms appear driven by disease behaviour with longitudinal scores highlighting a greater psychological burden enduring in those with persisting disease activity at follow up appointments. Whilst the IBD standards emphasise the importance of holistic psychological assessment at IBD diagnosis, separating psychiatric illness from a psychological response to severe physical symptoms is not possible at first presentation, with prompt establishment of disease control through shared treatment plans that prioritise health related quality of life the key first step to be taken (12, 34). In those with persisting disability and psychological disease burden over and above other markers of disease activity, targeted psychological intervention might then be considered, as has been shown to be effective in those with established disease (10, 35, 36). Though the strength of specific IBD disk cut-offs for predicting moderate-severe depression and anxiety symptoms fell in our underpowered post-treatment cohort, IBD disk *‘Emotions’* scores and HADS scores remained strongly linked. In our work, patients identified with persisting moderate depression or anxiety symptoms did not receive targeted intervention, but this data will be used to support service expansion to facilitate such measures being available in future.

Though longer symptom durations prior to diagnosis associated with increased psychological symptoms, a pre-existing MHD was not associated with delayed diagnosis in our cohort. The framework of our study does not allow for the contribution of recall bias to these differences. However, patients with pre-existing MHDs were more likely to receive courses of oral corticosteroids, despite being no more likely to progress to advanced therapies or have ongoing disease activity after a year of treatment. Whilst this finding has been previously noted in active IBD and may relate to increased service utilisation amongst these patients, it remains noteworthy given the long-established potential for steroids to cause neuropsychiatric complications (37–40).

The difference in size of the cohorts for each analysis, in particular the longitudinal cohort being less than half the size of the overall cohort (and consequently underpowered for validation) is a key limitation. Furthermore, all scores are also derived from a single IBD centre and whilst patients were managed by a single MDT, post diagnosis treatment was not protocolised. Additionally, whilst data was gathered regarding pre-existing MHD, no data on other confounding socioeconomic factors were collected. Modelling relating to psychological burden was adjusted for baseline characteristics, but this was not the case in analyses relating to 12-month outcome, increasing the risk of uncontrolled confounding.

Utilisation of the IBD disk is not time consuming and whilst we opted to complete it in the clinic room given our pre-diagnosis setting, it can be completed electronically or in the waiting room prior to consultation (41). Implementing it as a PROM at IBD presentation can help in the early identification of those who are likely to require more aggressive IBD treatment to obtain disease control. Moreover, it can reliably detect clinically relevant depressive symptoms. Whilst these may represent a reaction to physical symptoms at IBD presentation, use of the IBD disk during longitudinal follow-up may help identify those with a persisting psychological symptom burden beyond that attributable to disease activity alone.

## Data Availability

The raw data supporting the conclusions of this article will be made available by the authors, without undue reservation.

## References

[1] Neuendorf R Harding A Stello N Hanes D Wahbeh H. Depression and anxiety in patients with Inflammatory Bowel Disease: A systematic review. J Psychosom Res. (2016) 87:70–80. doi: 10.1016/j.jpsychores.2016.06.001, PMID: 27411754

[2] Barberio B Zamani M Black CJ Savarino EV Ford AC. Prevalence of symptoms of anxiety and depression in patients with inflammatory bowel disease: a systematic review and meta-analysis. Lancet Gastroenterol Hepatol. (2021) 6:359–70. doi: 10.1016/S2468-1253(21)00014-5, PMID: 33721557

[3] McManus S Bebbington PE Jenkins R Brugha T. Mental health and wellbeing in England: The Adult Psychiatric Morbidity Survey 2014. NHS Digital. (2016). Available online at: https://files.digital.nhs.uk/pdf/q/3/mental_health_and_wellbeing_in_england_full_report.pdf (Accessed May 28 2025).

[4] Umar N King D Chandan JS Bhala N Nirantharakumar K Adderley N. The association between inflammatory bowel disease and mental ill health: a retrospective cohort study using data from UK primary care. Alimentary Pharmacol Ther. (2022) 56:814–22. doi: 10.1111/apt.17110, PMID: 35770611

[5] Cooney R Tang D Barrett K Russell RK. Children and young adults with inflammatory bowel disease have an increased incidence and risk of developing mental health conditions: A UK population-based cohort study. Inflammatory Bowel Dis. (2024) 30:1264–73. doi: 10.1093/ibd/izad169, PMID: 37603846 PMC11291622

[6] Massironi S Pigoni A Vegni EAM Keefer L Dubinsky MC Brambilla P. The burden of psychiatric manifestations in inflammatory bowel diseases: A systematic review with meta-analysis. Inflammatory Bowel Dis. (2025) 31:1441–59. doi: 10.1093/ibd/izae206, PMID: 39270637

[7] Fairbrass KM Lovatt J Barberio B Yuan Y Gracie DJ Ford AC. Bidirectional brain–gut axis effects influence mood and prognosis in IBD: a systematic review and meta-analysis. Gut. (2022) 71:1773–80. doi: 10.1136/gutjnl-2021-325985, PMID: 34725197

[8] Sauk JS Ryu HJ Labus JS Khandadash A Ahdoot A Lagishetty V. High perceived stress is associated with increased risk of ulcerative colitis clinical flares. Clin Gastroenterol Hepatol. (2023) 21:741–9. doi: 10.1016/j.cgh.2022.07.025, PMID: 35952942

[9] Irving P Barrett K Nijher M de Lusignan S. Prevalence of depression and anxiety in people with inflammatory bowel disease and associated healthcare use: population-based cohort study. Evidence Based Ment Health. (2021) 24:102–9. doi: 10.1136/ebmental-2020-300223, PMID: 33785498 PMC8311072

[10] Eccles JA Ascott A McGeer R Hills E St.Clair Jones A Page LA. Inflammatory bowel disease psychological support pilot reduces inflammatory bowel disease symptoms and improves psychological wellbeing. Frontline Gastroenterol. (2021) 12:154–7. doi: 10.1136/flgastro-2019-101323, PMID: 33613949 PMC7873548

[11] Engel K Homsi M Suzuki R Helvie K Adler J Plonka C. Newly diagnosed patients with inflammatory bowel disease: the relationship between perceived psychological support, health-related quality of life, and disease activity. Health Equity. (2021) 5:42–8. doi: 10.1089/heq.2020.0053, PMID: 33681688 PMC7929923

[12] Kapasi R Glatter J Lamb CA Acheson AG Andrews C Arnott ID. Consensus standards of healthcare for adults and children with inflammatory bowel disease in the UK. Frontline Gastroenterol. (2020) 11:178–87. doi: 10.1136/flgastro-2019-101260, PMID: 32419908 PMC7223296

[13] Kok KB Byrne P Ibarra AR Martin P Rampton DS. Understanding and managing psychological disorders in patients with inflammatory bowel disease: a practical guide. Frontline Gastroenterol. (2023) 14:78–86. doi: 10.1136/flgastro-2022-102094, PMID: 36561780 PMC9763641

[14] Beekman E Verhagen A. Clinimetrics: hospital anxiety and depression scale. J Physiother. (2018) 64:198. doi: 10.1016/j.jphys.2018.04.003, PMID: 29895416

[15] Ghosh S Louis E Beaugerie L Bossuyt P Bouguen G Bourreille L. Development of the IBD disk: A visual self-administered tool for assessing disability in inflammatory bowel diseases. Inflammation Bowel Dis. (2017) 23:333–40. doi: 10.1097/MIB.0000000000001033, PMID: 28146002 PMC5319390

[16] Peyrin-Biroulet L Cieza A Sandborn WJ Coenen M Chowers Y Hibi T. Development of the first disability index for inflammatory bowel disease based on the international classification of functioning, disability and health. Gut. (2012) 61:241–7. doi: 10.1136/gutjnl-2011-300049, PMID: 21646246 PMC3245899

[17] Tadbiri S Nachury M Bouhnik Y Serrero M Hebuterne X Roblin X. The IBD-disk is a reliable tool to assess the daily-life burden of patients with inflammatory bowel disease. J Crohns Colitis. (2021) 15:766–73. doi: 10.1093/ecco-jcc/jjaa244, PMID: 33246337

[18] Le Berre C Flamant M Bouguen G Siproudhis L Dewitte M Dib N. VALIDation of the IBD-disk instrument for assessing disability in inflammatory bowel diseases in a french cohort: the VALIDate study. J Crohns Colitis. (2020) 14:1512–23. doi: 10.1093/ecco-jcc/jjaa100, PMID: 32417910

[19] Katsaros M Kalogirou M Katsoula A Paschos P Karabatsou S Tsionis T. P370 Correlation of the IBD Disk with intestinal ultrasound in patients with inflammatory bowel disease. J Crohn’s Colitis. (2023) 17:i499–500. doi: 10.1093/ecco-jcc/jjac190.0500

[20] Bjelland I Dahl AA Haug TT Neckelmann D. The validity of the Hospital Anxiety and Depression Scale: An updated literature review. J Psychosomatic Res. (2002) 52:69–77. doi: 10.1016/S0022-3999(01)00296-3, PMID: 11832252

[21] Wu Y Levis B Sun Y He C Krishnan A Neupane D. Accuracy of the Hospital Anxiety and Depression Scale Depression subscale (HADS-D) to screen for major depression: systematic review and individual participant data meta-analysis. BMJ. (2021) 373:n972. doi: 10.1136/bmj.n972, PMID: 33972268 PMC8107836

[22] Maaser C Sturm A Vavricka SR Kucharzik T Fiorino G Annese V. ECCO-ESGAR Guideline for Diagnostic Assessment in IBD Part 1: Initial diagnosis, monitoring of known IBD, detection of complications. J Crohns Colitis. (2019) 13:144–64. doi: 10.1093/ecco-jcc/jjy113, PMID: 30137275

[23] JASP Team. JASP (Version 0.18.3)[Computer software] (2024). Available online at: https://jasp-stats.org/ (Accessed August 28, 2024).

[24] The Jamovi Project. Jamovi (version 2.6.26)[Computer software] . Available online at: https://www.jamovi.org (Accessed May 28, 2025).

[25] Thiele C Hirschfeld G. cutpointr: Improved Estimation and Validation of Optimal Cutpoints in R. J Stat Softw. (2021) 98:1–27. doi: 10.18637/jss.v098.i11

[26] Robin X Turck N Hainard A Tiberti N Lisacek F Sanchez JC. pROC: an open-source package for R and S+ to analyze and compare ROC curves. BMC Bioinf. (2011) 12:77. doi: 10.1186/1471-2105-12-77, PMID: 21414208 PMC3068975

[27] Fox J Weisberg S. An R Companion to Applied Regression. 3rd ed. Thousand Oaks CA: Sage (2019). Available online at: https://www.john-fox.ca/Companion/.

[28] Massazza A Kienzler H Al-Mitwalli S Tamimi N Giacaman R. The association between uncertainty and mental health: a scoping review of the quantitative literature. J Ment Health. (2022) 32:480–91. doi: 10.1080/09638237.2021.2022620, PMID: 35014927

[29] Shafer LA Walker JR Chhibba T Ivekovic M Singh H Targownik LE. Independent validation of a self-report version of the IBD Disability Index (IBDDI) in a population-based cohort of IBD patients. Inflammation Bowel Dis. (2018) 24:766–74. doi: 10.1093/ibd/izx063, PMID: 29554260

[30] Malmborg P Everhov AH Soderling J Ludvigsson JF Bruze G Olen O. Earnings during adulthood in patients with childhood-onset inflammatory bowel disease: a nationwide population-based cohort study. Alim Pharmacol Ther. (2022) 56:1007–17. doi: 10.1111/apt.17148, PMID: 35916469 PMC9544615

[31] Storan D McDermott E Moloney J Keenen L Stack R Sheridan J. Inflammatory Bowel Disease Disability Index is a valid and reliable measure of disability in an English-speaking hospital practice and predicts long-term requirement for treatment escalation. Frontline Gastroenterol. (2024) 15:130–6. doi: 10.1136/flgastro-2023-102428, PMID: 38486665 PMC10935531

[32] Attauabi M Madsen GM Bendtsen F Seidelin JB Burisch J. Multidimensional patient-reported outcomes and quality of life at diagnosis of IBD: A population-based inception cohort study. Clin Gastroenterol Hepatol. (2025) 23:1418–27. doi: 10.1016/j.cgh.2024.08.047, PMID: 39461459

[33] Turner D Ricciuto A Lewis A International Organization for the Study of IBD. STRIDE-II: an update on the Selecting Therapeutic Targets in Inflammatory Bowel Disease (STRIDE) initiative of the International Organization for the Study of IBD (IOIBD): determining therapeutic goals for treat-to-target strategies in IBD. Gastroenterology. (2021) 160:1570–83. doi: 10.1053/j.gastro.2020.12.031, PMID: 33359090

[34] Fanizzi F D’Amico F Peyrin-Biroulet L Danese S Dignass A. Treatment targets in IBD: is it time for new strategies? Best Pract Res Clin Gastroenterol. (2025) 77:101990. doi: 10.1016/j.bpg.2025.101990, PMID: 40769616

[35] Tse CS Hunt MG Brown LA Lewis JD. Inflammatory bowel diseases-related disability: risk factors, outcomes, and interventions. Inflammatory Bowel Dis. (2024) 30:501–7. doi: 10.1093/ibd/izad182, PMID: 37603844

[36] Regueiro M Click B Anderson A Shrank W Kogan J McAnallen S. Reduced unplanned care and disease activity and increased quality of life after patient enrollment in an inflammatory bowel disease medical home. Clin Gastroenterol Hepatol. (2018) 16:1777–85. doi: 10.1016/j.cgh.2018.04.007, PMID: 29654918 PMC6185823

[37] Fairbrass KM Gracie DJ Ford AC. Relative contribution of disease activity and psychological health to prognosis of inflammatory bowel disease during 6.5 years of longitudinal Follow-Up. Gastroenterology. (2022) 163:190–203.e5. doi: 10.1053/j.gastro.2022.03.014, PMID: 35339461

[38] Graff LA Walker JR Bernstein CN. Depression and anxiety in inflammatory bowel disease: A review of comorbidity and management. Inflammatory Bowel Diseases. (2009) 15:1105–18. doi: 10.1002/ibd.20873, PMID: 19161177

[39] Feuerstein JD Rubin DT Aberra FN Yarur AJ Malter L. Appropriate use and complications of corticosteroids in inflammatory bowel disease: A comprehensive review. Clin Gastroenterol Hepatol. (2025) S1542-3565(25)00535-X. doi: 10.1016/j.cgh.2025.05.019, PMID: 40588110

[40] Hill E Nguyen NH Qian AS Patel S Chen PL Tse CS. Impact of comorbid psychiatric disorders on healthcare utilization in patients with inflammatory bowel disease: a nationally representative cohort study. Dig Dis Sci. (2022) 67:4373–81. doi: 10.1007/s10620-022-07505-9, PMID: 35503486

[41] Sharma N Savelkoul E Shah A De Silva S Pattni S Iaccuci M. A multicenter study of patient acceptability of the IBD disk tool and patient-reported disabilities. Dig Dis Sci. (2022) 67:457–62. doi: 10.1007/s10620-021-06893-8, PMID: 33721160

