# Supplementary material for: The IBD-disk accurately assesses disability and psychological burden at IBD diagnosis and predicts adverse outcomes in both UC and Crohn’s disease during the first year of treatment: a prospective observational cohort study
Source: Front Gastroenterol (Lausanne). 2025 Sep 11;4:1642061. doi: 10.3389/fgstr.2025.1642061 (PMC12952321; doi:10.3389/fgstr.2025.1642061)
Supplement: Supplementary file 1 [file Table1.docx]

*Criteria contributing to disease activity assessments at follow up visits in patients with UC and Crohn’s disease.*

| **Grading criteria** | **UC** | **Crohn’s** |
| --- | --- | --- |
| Clinical | Partial Mayo <3 | HBI <5 |
|  | No individual score >1 |  |
|  | No steroids or treatment escalation planned or undertaken within 3 months | No steroids or treatment escalation planned or undertaken within 3 months |
| Biochemical | CRP <5 AND FCP <150 | CRP <5 AND FCP <150 |
| Endoscopic or radiological | Mayo 0 or UCEIS ≤1 | SESCD <3 |
|  |  | **OR** |
|  |  | No active disease on imaging |
