# Supplementary material for: The IBD-disk accurately assesses disability and psychological burden at IBD diagnosis and predicts adverse outcomes in both UC and Crohn’s disease during the first year of treatment: a prospective observational cohort study
Source: Front Gastroenterol (Lausanne). 2025 Sep 11;4:1642061. doi: 10.3389/fgstr.2025.1642061 (PMC12952321; doi:10.3389/fgstr.2025.1642061)
Supplement: Supplementary file 2 [file DataSheet1.pdf]

IBD Disk

Questionnaire and scoring disk

Please fill in this questionnaire and give to the doctor at your appointment. We will use this information to try and improve your care

Which bowel disease do you have?

☐ Crohn's disease
☐ Ulcerative colitis (UC)
☐ I don't know

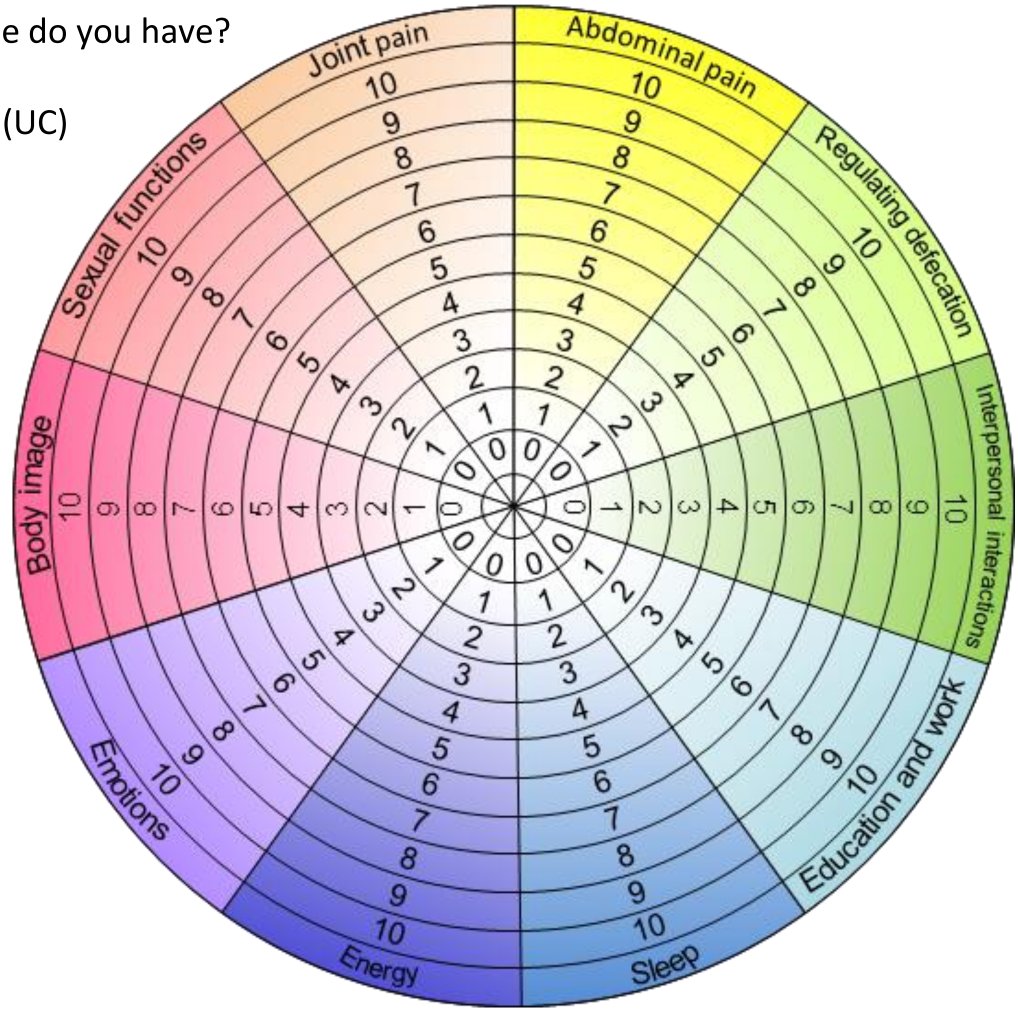

In the last week, because of my Crohn's disease or ulcerative colitis:

Score each statement on a scale of 0 to 10 and circle your score on the colored disc

1 2 3 4 5 6 7 8 9 10

Absolutely disagree — Neither agree or disagree — Absolutely agree

Abdominal pain	I have aches or pains in my stomach or abdomen
Regulating defecation	I have had difficulty coordinating and managing defecation, including choosing and getting to an appropriate place for defecation and cleaning myself afterwards
Interpersonal interactions	I have had difficulty with personal relationships and/or difficulty participating in the community
Education and work	I have had difficulty with school or studying activities, and/or difficulty with work or household activities
Sleep	I have had difficulty sleeping, such as falling asleep, waking up frequently during the night or waking up too early in the morning
Energy	I have not felt rested and refreshed during the day, and have felt tired and without energy
Emotions	I have felt sad, low or depressed, and/or worried or anxious
Body image	I have not liked the way my body or body parts look
Sexual functions	I have had difficulty with the mental and/or physical aspects of sex
Joint pain	I have had pains in the joints of my body
